# Comparison of Quantitative Techniques including Xpert MTB/RIF to Evaluate Mycobacterial Burden

**DOI:** 10.1371/journal.pone.0028815

**Published:** 2011-12-22

**Authors:** Richard N. van Zyl-Smit, Anke Binder, Richard Meldau, Hridesh Mishra, Patricia L. Semple, Grant Theron, Jonathan Peter, Andrew Whitelaw, Suren K. Sharma, Robin Warren, Eric D. Bateman, Keertan Dheda

**Affiliations:** 1 Lung Infection and Immunity Unit, Division of Pulmonology and UCT Lung Institute, Department of Medicine, University of Cape Town, Cape Town, South Africa; 2 Department of Medicine, All India Institute of Medical Sciences, New Delhi, India; 3 Division of Medical Microbiology, University of Cape Town, Cape Town, South Africa; 4 DST/NRF Centre of Excellence for Biomedical TB Research/MRC Centre for Molecular and Cellular Biology, Stellenbosch University, Stellenbosch, South Africa; 5 Institute of Infectious Diseases and Molecular Medicine, University of Cape Town, Cape Town, South Africa; 6 Department of Infection, University College London Medical School, London, United Kingdom; San Francisco General Hospital, University of California San Francisco, United States of America

## Abstract

**Introduction:**

Accurate quantification of mycobacterial load is important for the evaluation of patient infectiousness, disease severity and monitoring treatment response in human and *in-vitro* laboratory models of disease. We hypothesized that newer techniques would perform as well as solid media culture to quantify mycobacterial burden in laboratory specimens.

**Methods:**

We compared the turn-around-time, detection-threshold, dynamic range, reproducibility, relative discriminative ability, of 4 mycobacterial load determination techniques: automated liquid culture (BACTEC-MGIT-960), [^3^H]-uracil incorporation assays, luciferase-reporter construct bioluminescence, and quantitative PCR(Xpert -MTB/RIF) using serial dilutions of *Mycobacterium bovis* and *Mycobacterium tuberculosis* H37RV. Mycobacterial colony-forming-units(CFU) using 7H10-Middlebrook solid media served as the reference standard.

**Results:**

All 4 assays correlated well with the reference standard, however, bioluminescence and uracil assays had a detection threshold ≥1×10^3^ organisms. By contrast, BACTEC-MGIT-960 liquid culture, although only providing results in days, was user-friendly, had the lowest detection threshold (<10 organisms), the greatest discriminative ability (1 vs. 10 organisms; p = 0.02), and the best reproducibility (coefficient of variance of 2% vs. 38% compared to uracil incorporation; p = 0.02). Xpert-MTB/RIF correlated well with mycobacterial load, had a rapid turn-around-time (<2 hours), was user friendly, but had a detection limit of ∼100 organisms.

**Conclusions:**

Choosing a technique to quantify mycobacterial burden for laboratory or clinical research depends on availability of resources and the question being addressed. Automated liquid culture has good discriminative ability and low detection threshold but results are only obtained in days. Xpert MTB/RIF provides rapid quantification of mycobacterial burden, but has a poorer discrimination and detection threshold.

## Introduction

Determining mycobacterial burden is a basic requirement for many laboratory and clinical studies, including those assessing disease severity and examining the efficacy of new therapies and interventions for tuberculosis (TB) control [1], [2], [3], all of which have now become urgent with the emerging public health threat of multidrug and extensively drug resistant TB [4], [5]. In addition, mycobacterial burden, usually assessed as grades of smear positivity (scanty, 1+2+, and 3+), is used to evaluate the infectiousness of cases in the context of public health contact tracing and screening [6], [7]. In translational research the need to accurately detect changes in *M. tuberculosis* burden is fundamental to the study of biologically meaningful immunological pathways, and drug and vaccine development in both human and murine models of disease. The latter include early bacteriocidal activity (EBA) studies related to drug development and evaluating vaccine efficacy in murine models [8], [9], [10], [11]. Although several techniques for determining mycobacterial burden exist, each is associated with significant limitations such as inaccuracy, turn-around-time, limited reproducibility, cost, methodological complexity, relative discriminative ability and detection threshold.

Culture on solid media using colony-forming units (CFU), is widely considered to be the gold standard for determining the number of viable organisms in a specimen or experimental condition, but is labor-intensive and has a long turn-around-time [12], [13]. Alternative techniques include the incorporation of tritiated uracil into mycobacterial DNA, bioluminescence assays that use a reporter construct, quantitative real-time polymerase chain reactions (PCR), and time to positivity (TTP) in automated liquid culture systems (BACTEC Mycobacterial Growth Indicator Tube (MGIT) 960] [14]. Each of the later has its own set of performance characteristics that determine its suitability for different applications.

More recently, newer technologies such as the Xpert MTB/RIF system (Cepheid, Sunnyvale, USA) have been developed for the rapid detection of TB using clinical samples. However PCR methods have been limited by their inability to distinguish viable from degraded organisms. Whilst detecting TB-specific mRNA from viable organisms is a potential solution, like PCR [15], [16], real time PCR it is technically demanding [17]. However, Xpert MTB/RIF has the potential to circumvent this problem as contaminating extracellular debris is removed in an intermediary step by washing, DNA from intact organisms trapped in a mesh is amplified by PCR [18], [19]. However, its quantitative accuracy has not yet been compared with that of automated culture, uracil incorporation and bioluminescence techniques.

We hypothesized that newer automated mycobacterial load determination techniques perform as well as traditional measures. We therefore compared the performance characteristics of 4 quantitative techniques by evaluating turn-around-time, detection threshold, dynamic range, reproducibility and quantitative discriminative ability, with CFU on solid media as the reference standard.

## Methods

**Figure 1:**
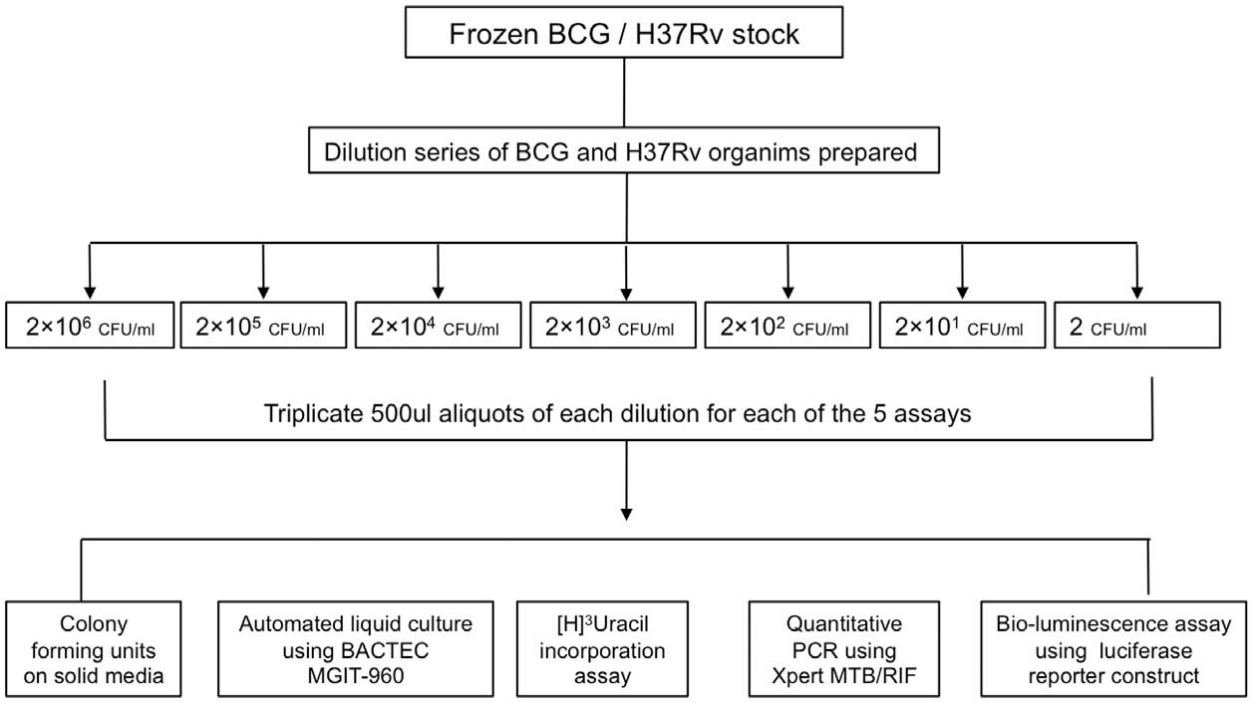
Preparation of Mycobacterial dilutions for load determination assays. A three-milliliter volume of frozen stock was diluted to a working suspension of 2×10^6^ CFU/ml. From this stock 5 serial dilutions were prepared ranging from 2 to 2×10^6^ CFU/ml. From these dilutions, 15 aliquots of organisms were prepared containing 1 to 1×10^6^ CFU in 500 ul. Three aliquots of each dilution were used in each of the 5-mycobacterial load determination assays as described in the methods section. PCR-polymerase chain reaction, MGIT mycobacterial growth indicator tube, CFU-colony forming unit.

Both BCG and H37RV luciferase reporter constructs (pSMT1 luciferase) [20] were used for all assays (gift of Muazaam Jacobs from the Institute for Infectious Diseases and Molecular Medicine University of Cape Town). Serial dilutions were prepared (aliquots ranging from 1 to 1×10^6^ CFU per 500 ul) in sterile phosphate buffer solution (PBS) from 3 ml of frozen stock for each strain. Three vials of each dilution were provided for the five predetermined assays. In addition, all dilutions were inoculated onto solid media to confirm the number of CFUs at each dilution (Figure 1).

### Solid Culture (reference standard)

Aliquots of 1, 10 and 100 CFU were plated in 6 replicates of 10 µl on 7h10 Middlebrook agar enriched with OADC. Plates were sealed in airtight bags and incubated at 37°C. Colonies were counted daily between days 7 and 14 using an inverted light microscope. The colony counts were over two days by two readers.

### Liquid culture

A 500 µl aliquot of PBS containing the specified number of organisms was injected into pre-prepared mycobacterial growth indicator tubes (MGITs) (Becton Dickinson, Sparks, Maryland), and the MGITs then incubated using the BACTEC-MGIT-960 automated culture system, which monitors the tubes for the presence of growth on a constant basis. Each dilution was prepared in triplicate and blinded to the MGIT operator. Time to positivity was recorded by instrument as the time between start of incubation and detection of growth.

### Luminescence assay

Luminescence was measured using a Modulus™ Microplate Multimode Luminometer (Turner). 25 µl of 1% (v/v) n-decyl aldehyde (Sigma), was injected into each well containing serial dilutions (1∶10 with 0.25% Tween/PBS) to a final volume of 125 µl. The results were read using a 0.5 s delay and a 0.5 s integration time and were expressed as relative light units (RLU).

### Xpert MTB/RIF PCR

A 500 ul aliquot of H37RV and BCG was treated with sample buffer at a ratio of 1∶3 supplied by the manufacturer as recommended. The 2 ml mixture was later transferred into an Xpert MTB/RIF cartridge. The cartridge was loaded into the Gene Xpert IV instrument and the automated procedure started. Results and C_T_ values of probes were obtained using the software (Xpert MTB/RIF version 2.0).

### Uracil incorporation

200 µl of each dilution was pipetted into 6 wells of 4 separate U-bottomed 96 well plates and 50 ul of ^3^H uracil (final concentration of 1 µCi/well) was added to each well. The plates were incubated for 24 hours at 37°C in a 5% CO_2_ humidified chamber and harvested onto fiberglass filter mats. The discs were placed into scintillation bottles containing 1 ml of Quicksafe (Zinnser Analytic, Frankfurt, Germany) and the amount of incorporated tritiated uridine determined using a liquid scintillation counter and reported as counts per minute (CPM).

### Determination of performance characteristics

#### Turn-around-time

The turnaround time was defined as the time taken from the start of each assay after preparation of the aliquots until a bacterial load determination was possible (either automated output or colony counting).

#### Detection threshold

The detection threshold was defined as the lowest colony number detected by the assay: The result from 2 out of 3 MGIT bottles, 2 out of 3 Xpert MTB/RIF cartridges or >50% of CFU/RLU/CPM replicates for a particular dilution were as the reliable lower limit of detection for comparative purposes.

#### Discriminative ability

The ability of the assay to detect a difference between serial dilutions was assessed using a 1-way ANOVA with correction for multiple comparisons (Tukey). GraphPad Prism software (version 5.00, GraphPad Software, San Diego California USA, www.graphpad.com).

#### Reproducibility

Reproducibility of each assay was determined by calculating the coefficient of variance (SD/mean) across all dilutions. To compare reproducibility between assays the mean coefficient of variance for BCG dilutions was calculated for each assay.

## Results

A comparative overview of all the mycobacterial load assays is summarised in Table 1. The performance characteristics of each assay are detailed separately.

**Table 1:** Performance characteristics of individual techniques used to quantify mycobacterial burden.

Assay	Turn-around time	Detection threshold (CFU; 1 CFU = 1 organism)	Dynamic range*	Reproducibility (Coefficient of Variance)+
**Culture on 7H10 solid media**	Days to weeks	1 CFU	Wide provided appropriate dilutions are made.	Fair (22%)
**Liquid culture using MGIT 960 system**	Days to weeks	1–10 CFU	Excellent from 1 to 1×10^6^ CFU	Good (2%)
**Tritiated uracil assay**	24 hours	1000 CFU	Poor below 1×10^3^ CFU but able to detect dilutions of up to 1×10^6^ CFU	Poor (38%)
**Luminescence assay using reporter construct**	<2 hours	100 CFU	Poor below 1×10^3^ CFU but able to detect dilutions of up to 1×10^6^ CFU	Fair (19%)
**PCR using** Xpert MTB/RIF	2 hours	100 CFU	Good at ranges between 1×10^2^ and 1×10^6^ CFU	Good (3%)

*All experiments (apart from solid culture) require a standard curve to calculate the CFUs relative to the assay readout.

+ Reproducibility was determined by the mean coefficient of variance for the specific readout (CFU, RLU etc.) across all dilutions using the BCG-specific experiments.

### Solid media culture

**Figure 2:**
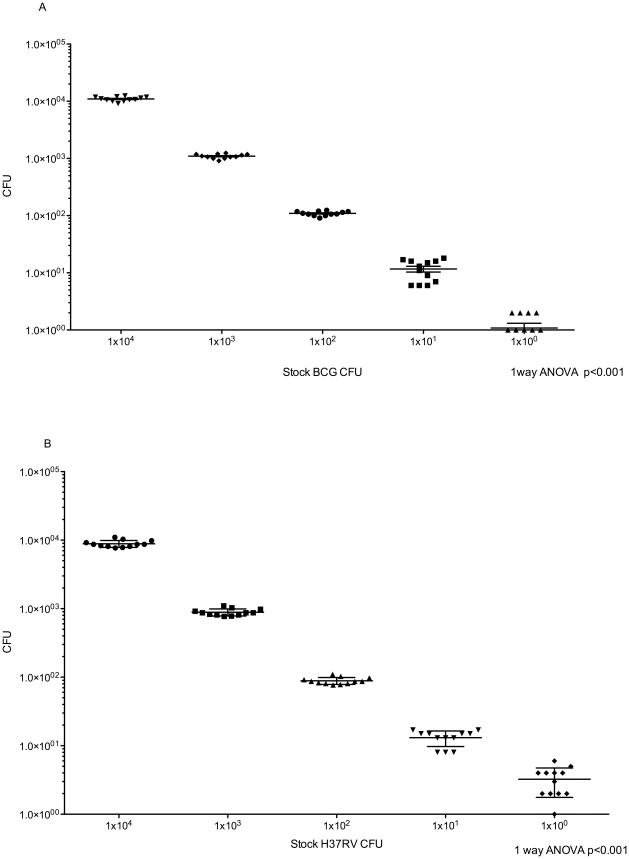
Mycobacterial load determination using solid media (Middlebrook 7H10). Serial dilutions of stock BCG (A) and H37RV (B) are represented on the X-axis with the calculated CFU on the Y-axis (log scale).

Although technically simple to perform, plating for CFU was time consuming and accuracy and reproducibility are affected by pipetting skills as well visual counting of colonies. Counting of CFUs and determination of mycobacterial load was possible approximately 10–14 days after plating (Figure 2). Determination of the CFU count was limited by the visual ability to accurately count organisms and thus only dilutions of 1, 1×10^1^ and 1×10^2^ CFU were used for plating on 7H10 agar. The coefficient of variance across the three dilutions ranges was 22%.

### Automated liquid culture

**Figure 3:**
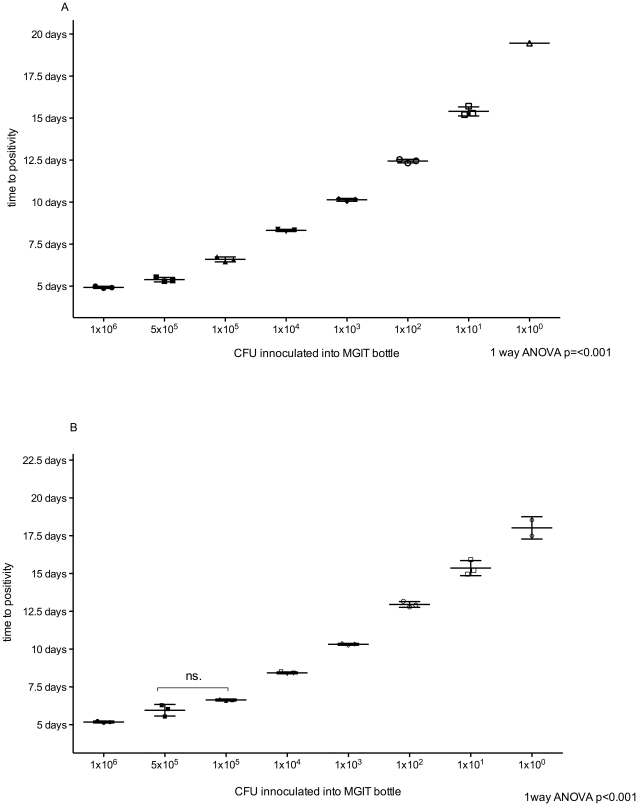
Automated liquid culture (using BACTEC MGIT 960) time to positivity. Serial dilutions of stock BCG (A) and H37RV (B) are represented on the X-axis with automated positive detection time (time to positivity) on the Y-axis.

The turnaround time for the BACTEC-MGIT-960 system was defined as time to positivity (TTP) and ranged from 117 hours (∼5 days) to 467 hours (∼19 days) for BCG and 123 hours (∼5 days) to 528 hours (∼22 days) for H37RV (dilution range 1×10^6^ to 1×10^0^ CFU; Figure 3) The lower limit of detection was less than 10 CFU (1 CFU detected in 3/3 BCG bottles and 2/3 H37RV bottles). Reproducibility of the BACTEC-MGIT-960 system was excellent as most replicates became positive within a few hours of each other at all dilutions (coefficient of variance = 2%). The range of detection was from 1 to 1×10^6^ CFU. BACTEC-MGIT-960 was able to detect differences in mycobacterial load as small as 1×10^2^ organisms at low concentrations and 1×10^5^ at higher concentrations.

### Luminescence assay

**Figure 4:**
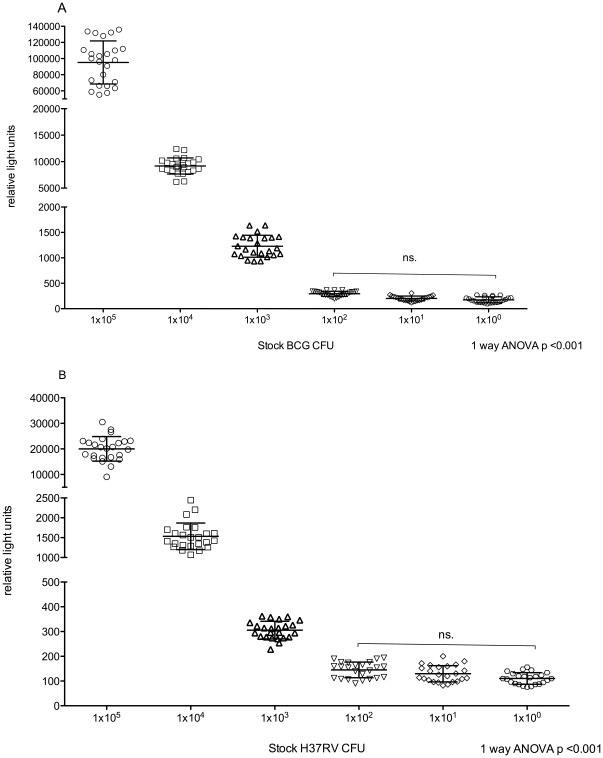
Luminescence assay (using luminescent reporter construct) relative light units. Serial dilutions of stock BCG (A) and H37RV (B) are represented on the X-axis with the relative light unit output on the Y-axis.

The lower limit of detection was 1×10^2^ CFU as no discrimination was possible below 100 organisms. Discrimination between was possible at ranges of 1×10^2^ to 1×10^5^ CFU (Figure 4). Reproducibility was comparable to CFU with a coefficient of variance of 19%. Mycobacterial load determination was potentially available within minutes of RLU determination using a pre-prepared standard curve of RLU vs. CFU.

### Uracil

**Figure 5:**
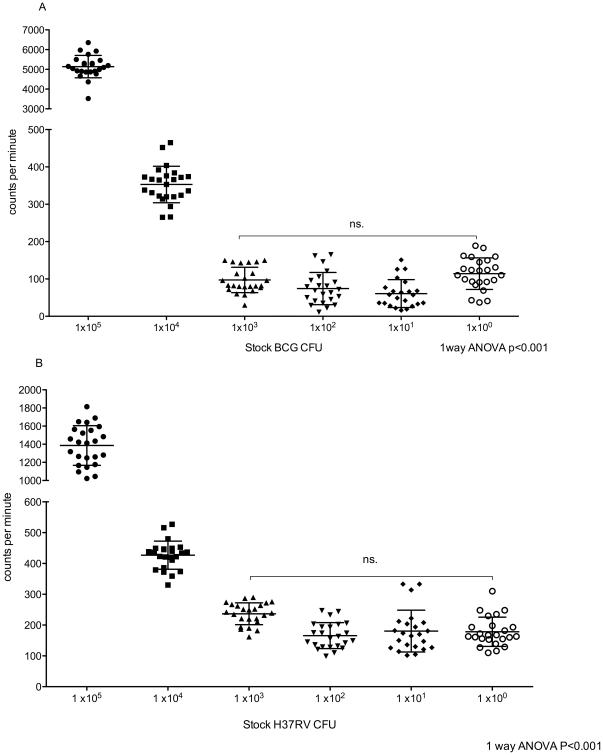
Uracil incorporation assay counts per minute (CPM). Serial dilutions of stock BCG (A) and H37RV (B) are represented on the X-axis with the calculated CPM on the Y-axis.

Uracil incorporation assays are complex and time consuming to perform although the time to acquiring a result was approximately 24 hours. The effective lower limit of detection was 1000 CFU as the tritiated uracil assay could detect but not discriminate between loads at ranges below 1×10^3^ CFU. However, it was effective at 1×10^3^ to 1×10^5^ CFU (Figure 5).

### PCR using the Xpert MTB/RIF system

**Figure 6:**
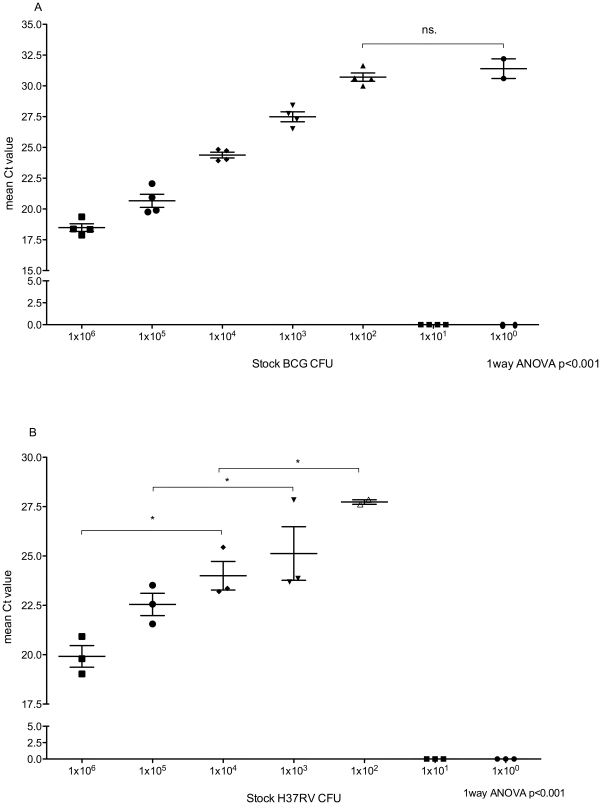
Automated PCR (using Xpert MTB/RIF) changes in cycle threshold (C_T_). Serial dilutions of stock BCG (A) and H37RV (B) are represented on the X-axis with the C_T_ value on the Y-axis. For BCG, C_T_ values were significantly different between serial dilutions (following correcting for multiple comparisons) except where indicated. For H37Rv, * indicate significant difference in C_T_ values between replicates (following correcting for multiple comparisons).

The automated real time MTB/RIF assay using the Gene Xpert IV system was user friendly and minimal technical steps were required prior to inserting the cartridge into the machine. Xpert MTB/RIF results were available within 2 hours of the experiment using the Gene Xpert IV machine with 4 cartridge bays (only 4 samples could be run at once). BCG reproducibility was excellent (coefficient of variance 3%) and the automated PCR reliably detected 100 organisms (Figure 6). At the lowest dilution of 1 CFU, only 2 out of 3 cartridges were positive. The H37RV had greater variability in cycle threshold (C_T_) values as compared to BCG (coefficient of variance 6%) and did not detect less than 100 CFU. For the BCG experiments mean C_T_ values were statistically significant across all ranges thus providing excellent discriminative ability. Although the mean Ct values across the range of loads tested were statistically different for the H37RV experiments (1way ANOVA p<0.001), when correcting for multiple comparisons (Tukey), statistical significance was only present at a 2 log change in CFU i.e. 1×10^4^ vs. 1×10^6^.

## Discussion

Accurate determination of mycobacterial numbers is essential for evaluating infectivity, the efficacy of treatments and for many projects in translational research. However, there are limited reports comparing the performance of different mycobacterial load determination techniques. We evaluated several techniques and to our knowledge head-to-head comparison of several technologies has not been previously undertaken.

Our study has demonstrated that (i) Xpert MTB/RIF provides a rapid measure of mycobacterial burden above a threshold of ∼100 organisms/sample; (ii) TTP using MGIT 960, whilst accurate, with better discriminative ability of mycobacterial load (than Xpert MTB/RIF) and a detection threshold down to 1 CFU, has a substantially longer turn-around-time; (iii) bioluminescence and uracil incorporation assays are limited by lack of discrimination below 1000 organisms. Thus, none of these assays has ‘ideal’ performance characteristics and the selection of an assay has to based on the experimental question under study and details of the study design including the likely range of bacterial burden which is anticipated, need for rapid results, desired reproducibility, and available laboratory and financial resources. In resource-limited settings determination of CFU using solid media is likely to remain the method of choice as it is likely to be associated with the least cost.

Although PCR for mycobacterial load is not a novel technique, the Xpert MTB/RIF assay may overcome several of the known drawbacks of real-time PCR. Most notably, apart from the semi automation of the methods, DNA from degraded organisms is thought to be removed in the wash step and only intact organisms are retained in the cartridge mesh for the PCR step [19]. Our data supports this view as Xpert-related results correlated with those derived from solid culture CFU counts and thus intact organisms. Although the Xpert MTB/RIF assay detects intact organisms, it cannot distinguish viable from non-viable organisms and thus suffers from similar drawbacks to conventional NAATs and smear microscopy. All the other assays of mycobacterial burden examined require the organisms to be viable and for detection and hence are unable to detect the presence of intact but dead organisms. Our work was specifically undertaken to establish comparative utility of various techniques when performing in vitro laboratory studies using human cells and not for clinical studies. However, given its rapid turn-around-time and good correlation with mycobacterial burden studies are now required to evaluate the utility of Xpert MTB/RIF for monitoring treatment response, disease prognosis, and evaluating risk of disease transmission. Like with smear microscopy quantitative stratification is feasible. A recent study has shown that Xpert MTB/RIF cycle threshold correlates with organism load as defined by smear and MGIT time to positivity [21]. In this study the discriminative ability of Xpert to detect changes in organism load was suboptimal for H37RV compared to BCG. Larger studies including clinical isolates are required to verify this finding. Furthermore, the value of mycobacterial load, as determined by Xpert has yet to be demonstrated in the setting of a controlled clinical trial, particularly, to what extent the presence of dead organisms in sputum may confound the evaluation of treatment responses. Xpert has the added advantage of sensitivity as it confirms infection in a significant proportion of smear negative TB cases [21], [22] and controls for PCR inhibitors through an internal control.

Rapid turn-around-time is a further advantage of the Xpert MTB/RIF assay, which might be of value in drug development. For example, for providing ‘real time’ serial quantification of bacterial burden during early bacteriocidal activity studies. Its ability to detect quantitative changes in viable organisms will need to be evaluated as all PCR techniques will detect DNA from both live and dead organisms [23], [24]. By contrast, liquid culture results, although accurate, can take up to 6 weeks. However, the clinical value of rapid turnaround time will needs to weighed up against the potential greater cost and cost effectiveness studies will be required at a national implementation level. Thus, in laboratory studies while the rapid turnaround is highly attractive, it is likely to come at a significantly higher cost compared to solid culture. Another key drawback is the detection threshold of 100 organisms, which may be inadequate for experimental models where a low organism burden needs to be measured.

Liquid culture using the automated BACTEC 960 MGIT system is an attractive quantification technology for both clinical and laboratory studies. Already incorporated into EBA studies [12], [13] TTP has been well correlated with bacterial load [7], [13], [14]. Its user-friendly format, automation, high discriminative ability and low detection threshold (less than 10 organisms) makes it well suited to laboratory studies. The key drawbacks of liquid culture are the need to decontaminate clinical samples as MGIT cultures are prone to bacterial overgrowth, in addition to the slower turnaround time (days to weeks), compared to Xpert MTB/RIF and solid culture, respectively [25].

Bioluminescence and uracil incorporation assays both provide rapid turnaround time and are have been extensively used in laboratory studies [10], [26], [27]. They have limited application to clinical and public health studies as the findings are strain-specific and there is a higher risk of bacterial contamination with uracil incorporation assays. Both assays have limited discriminative ability below 1000 organisms/sample. Uracil incorporation assays offer little advantage over bioluminescence yet require significant additional infrastructure to accommodate the storage and disposal of radioactive waste. As with all the other “indirect” techniques of quantitative load determination, a standard curve would be required to calculate actual CFU counts if required.

There are several limitations to this study. The findings here pertain to *in vitro* laboratory work done with a specific strain and thus may not be generalizable to clinical strains or clinical samples where sample quality and inhibitors etc. may impact on findings. Smear microscopy was not used as a reference standard in this study as the primary focus was on laboratory settings where organism load below 100 000 organisms/ml are frequently used and smear looses sensitivity below this threshold. Moreover, we required a highly discriminative technique from a quantitation point of view (smear only provides broad categorical data i.e. 1+ or 2+ etc. with poor discriminatory value). Therefore we chose not to evaluate smear microscopy. Thus, because a single strain was used for all experiments the MGIT findings may also not be generalizable to clinical samples or different strains. Whilst Ct correlated well with mycobacterial burden, as shown in recent studies [21], to what extent it will correlate with risk of infection in contacts of index cases remains to be determined.

In summary, no single mycobacterial quantitative technique has ideal performance characteristics. Thus, the choice of assay will largely depend on the research context, study question, and the relative tradeoffs of cost versus turnaround time. Automated systems like MGIT are sensitive and have high discriminatory value but a long turnaround time. Xpert MTB/RIF is a good quantitative tool with rapid turn-around-time but its detection threshold was not as good as automated liquid culture. Although solid culture is the most labor-intensive it likely remains the cheapest option for highly discriminative quantification of mycobacterial load over a wide dynamic range. Cost effectiveness and clinical treatment follow up studies are still required for Xpert and MGIT.
